# A high-throughput, rapid mail-in program for XFEL chemical crystallography at the Linac Coherent Light Source

**DOI:** 10.1063/4.0001140

**Published:** 2025-10-27

**Authors:** Elyse A. Schriber, Daniel J. Rosenberg, Daniel W. Paley, Frederic Poitevin, Maggie C. Willson, Kelsey Banta, J. Nathan Hohman, Aaron Brewster

**Affiliations:** 1 SLAC National Accelerator Laboratory, Menlo Park, CA, USA; 2 Lawrence Berkeley National Laboratory, Berkeley, CA, USA; 3 University of Connecticut, Storrs, CT, USA

## Abstract

Small-molecule serial femtosecond crystallography (smSFX) at XFELs has become a reliable tool for determining structures of new, unknown materials from microcrystalline powders. The use of smSFX has had a considerable impact on metal-organic chalcogenolate (MOCHa) research, where smSFX derived structures have contributed new knowledge on the structure-function relationships in MOCHa compounds and have informed MOCHA synthetic efforts significantly. The vast demand in materials science and chemistry fields for structural techniques that produce high-quality, accurate structures from microcrystalline samples show the impact the smSFX technique can have on other compound classes. To expand smSFX capabilities to the larger global community, we have developed the world’s only mail-in program for XFEL chemical crystallography at the Linac Coherent Light Source, performing data collection as a service for outside sample submitters from the US and worldwide. The inaugural mail-in smSFX experiments identified four highly technologically relevant compound classes where smSFX will have the greatest impact. Metal-organic frameworks (MOFs), covalent-organic frameworks (COFs), inorganic covalent solids, and organic molecular crystals. Our automated sampled delivery and data collection methods allow us to collect data in a high-throughput manner and provide these user groups with high-quality, accurate structures. In one mail-in beamtime, we can impact research efforts on a global scale and contribute new science to multiple technologically relevant compound classes.

